# Supplementary material for: Machine-Learning Techniques for Feature Selection and Prediction of Mortality in Elderly CABG Patients
Source: Healthcare (Basel). 2021 May 7;9(5):547. doi: 10.3390/healthcare9050547 (PMC8151160; doi:10.3390/healthcare9050547)
Supplement: Supplementary file 1 [file healthcare-09-00547-s001.zip › healthcare-1171821-supplementary.pdf]

Appendix A. ICD-9-CM and ICD-10-CM code used for diagnosis in this study.

Variables	ICD_9_CM	ICD_10_CM
Ischemic stroke	433–434, 436, 437.1	I63- 64
Hypertension	401–405	I10–I14
Hyperlipidemia	272.0–272.4	E78
Cardiogenic shock	785.51	R57.0
Liver cirrhosis	571	K73.9, K73.0–K75.4, K74.1, K74.2, K74.5, K74.69
Diabetes mellitus (DM)	250	E10–E14
Atrial fibrillation (AF)	427.31	I48
Myocardial infarction (MI)	410–411	I21–I22
Acute coronary syndrome (ACS)	411.1 and 411.8	I20.0, I24.0, I24.8, I24.9
Transient ischemic attack (TIA)	435	G450
Congestive heart failure (CHF)	428.0, 428.1, 428.9	I50
Peripheral vascular disease (PVD)	249.7, 250.7, 443,447, 785.4	I70, I71, I73.9
Coronary artery disease (CAD)	410-414	I43, E78, I130.8, E78.4, I42.2, I27.2, I71.2, I42.1, I30.9, I71, I10, I33, I31.3, I26, I11, I34, I35.8, I07.1, Q25.3, I35I70, I25.1, I25.8, I25.6, I20.9, I24.5, I24.9, I54, I25
Chronic kidney disease (CKD)	585.4–585.5	N18.4, N18.5
Chronic obstructive pulmonary disease (COPD)	491–492, 494, 496	J40–J44 and J47
Renal disease	586, 585, 593.9	N18, N18.1–N18.5, N18.9
Intracranial bleeding		

Malignant dysrhythmia	4260, 426.12–426.13, 426.51–426.54, 4271, 4274, 427.41–427.42, 4275	I442, I441, I452, I453 I470, I472, I473, I4901, I4902
Acute pancreatitis	577.0	B252, K850, K851, K852, K853, K858, K859
Acute kidney failure	584, 584.5–584.9	N17, N17.0–N17.2, N178, N179
